# Exploring the impact of innovation guidance on user participation in online communities: A mixed methods investigation of cognitive and affective perspectives

**DOI:** 10.3389/fpsyg.2022.1011837

**Published:** 2022-09-28

**Authors:** Yang Li, Xiaona Gou, Haiqing Hu, Hongying Zhang

**Affiliations:** ^1^Business School, Shandong Normal University, Jinan, China; ^2^Meigu College, Shandong Normal University, Jinan, China

**Keywords:** online communities, user participation, innovation guidance, mental simulation, product type

## Abstract

In recent years, many online communities have launched opinion-gathering activities to promote user participation in innovation and improve the quality of new products. The current methods for online innovation activities can be divided into two categories: cognitive guidance and affective guidance. However, the studies on online communities have mainly focused on user engagement motivations, and little attention has been paid to investigating the impact and underlying mechanism of innovation guidance on user participation at the linguistic level. This study first collected secondary data from NetEase.com and conducted an econometric model to explore the impact of cognitive guidance and affective guidance on users’ participation in online innovation activities. Subsequently, we investigated the impact mechanism of different innovation guidance methods on user participation through two experiments, here by drawing on mental simulation theory. The experimental results showed that outcome simulation and process simulation imposed a dual mediating effect of innovation guidance on user participation. In addition, we also found that product types moderate the dual mediating effect of outcome simulation and process simulation. The findings can deepen and expand the research on user participation while providing practical implications for companies and platforms as they attempt to promote user participation in innovation activities.

## Introduction

Online communities have become the main communication platform between companies and users. Effective community operations are crucial for companies to obtain business value. Studies have found that online communities have positive effects on user loyalty (Habibi et al., 2016), word-of-mouth marketing (Munnukka et al., 2015), advertising (Zeng et al., 2017), and sales performance (Ho, 2015). In addition, online communities can help companies realize co-creation. In online communities, users can share important information with other users or companies, such as recommendations and product evaluations, which helps companies access market demand and improve product design and quality in a timely fashion (Thompson and Sinha, 2008). Xiaomi launched the MIUI community in 2010, encouraging users to participate in product R&D issues. Xiaomi officials will actively initiate or participate in user interactions, promoting product development and innovation while tailoring their products to suit consumer demand to the greatest extent, and receiving positive feedback from the market.

However, a common challenge faced by online communities is how to effectively obtain user opinions and motivate users to participate in innovative activities. Many communities (e.g., NetEase-LIFEASE Community, Haier-Smart Home Community, etc.) have launched innovation activities to collect users’ opinions, in which the platform first introduces and guides the activity and then invites users to share their opinions and suggestions within a specified period. For online community participation, motivation and guidance are critical. From the perspective of linguistic style, the innovation guidance provided by the platform can be broadly classified into two categories: cognitive guidance and affective guidance. When providing cognitive guidance, the platform first explains the background of the product and then provides different dimensions for users to provide feedback, such as *“Please give us your suggestion on our watch design. For example, what kind of dial design do you like? Which material for the strap do you want? Comment below, and we may feature your watch in our next product launch.”* When using affective guidance, the platform mainly uses contextualized context and emotional language, such as *“Home is where the heart is, but did you know that the things you have in your home can actually make you happier? There are thousands of appliances on the market designed to make life a little bit easier and bring some extra joy to your days. What’s your favorite? Please, leave your comment, and remember to give the reasons!”*

Cognition and affection are two pathways through which individuals process information, and they have been shown to impose significant effects on user behavior (Chen et al., 2016). However, the effect mechanisms of the two pathways are entirely different (D'arcy and Lowry, 2019). Although previous studies have provided some insights into the user motivation to participate in online communities (Jin et al., 2015), most of them have overlooked the antecedent of user participation, and innovation guidance. To fill this research gap, the current study proposes the first research question: *Do cognitive guidance and affective guidance significantly impact user participation in online innovation activities? What are the differences between these two methods in terms of their influence mechanism?*

In the marketing communication process, consumers usually imagine product-related imagery based on the communication message; this process is defined as mental simulation (Rather and Hollebeek, 2021; Zhong and Zhang, 2021). In the promotion of new products, mental simulation is a common marketing strategy sellers adopt to help customers learn and recognize products, reduce uncertainty, boost sales, and improve consumer evaluations (Zhao et al., 2011). Scholars have divided mental simulation into two categories: process simulation and outcome simulation (Pham and Taylor, 1999). Previous studies have found that these two types of mental simulation differ in their impact on consumer behavior: process simulation can boost behavioral intentions by enhancing users’ positive emotions, while outcome simulation can reduce psychological costs at the cognitive level and enhance purchase intentions (Pham and Taylor, 1999). Therefore, the present study argues that online communities can engage users in participating in innovative activities through cognitive and affective guidance, the influence mechanism of which is to stimulate users’ participation and information-sharing intention *via* evoking their mental simulation. To further verify the impact mechanism of different guidance methods, the present study puts forward the second research question: *Do process simulation and outcome simulation mediate the impact of affective guidance and cognitive guidance on user participation in online innovation activities?*

In addition, prior studies have found that product types can affect users’ value judgments, and users generally have different attitudes toward different types of products (Voss et al., 2003). In the field of information systems and marketing, many studies have been conducted based on the classification of hedonic products and utilitarian products (Drolet et al., 2007; Lim and Ang, 2008). Utilitarian products are based on functional, practical, cognitive, goal-oriented, and other attributes; these products can be household goods, office supplies, and so forth. Hedonic products refer to products based on attributes, such as experiences, emotion, pleasure, and esthetics, such as clothing, cosmetics, jewelry, and so forth (Dhar and Wertenbroch, 2000). It has been demonstrated that product types can moderate the information processes of online users (Islam et al., 2021). Therefore, the present study argues that cognitive guidance and affective guidance may result in different incentive effects on user participation in different products. To verify the moderating effect of product types, the current study proposes the third research question: *Do product types moderate the impact of different guidance methods on user participation in online innovation activities? That is, from the perspective of communities, should communities adopt different guidance methods to achieve better motivational effects for different product types?*

To advance this line of research, we investigated three aspects regarding the impact of innovation guidance on user participation in online innovation activities: main effect, mediating mechanism, and boundary condition. We first collected secondary data on 216 innovation activities from NetEase.com and used an econometric model to study the impact of cognitive guidance and affective guidance on user participation. We then used two random experiments to verify the mediating effect of mental simulation and moderating effect of product types. The results can deepen and improve the theoretical system of online community user behavior while providing management with inspiration for how companies can enhance the level of user participation and create business value.

The rest of the current paper is organized as follows: First, we review the literature related to our study. We then present the research hypothesis. Next, we describe the three substudies conducted to test the hypothesis. Finally, we discuss our main findings, theoretical contributions, and managerial implications.

## Literature review

### User participation in online communities

Online communities, also known as virtual communities, are a group of people with a common interest or shared purpose, whose interactions are governed by policies in the form of tacit assumptions, rituals, protocols, rules, and law and who use computer systems to support and mediate social interaction and facilitate a sense of togetherness (Preece, 2001; Tsai and Bagozzi, 2014). Through online communities, many companies actively invite users to participate in innovation activities for product design and improvement, which have created tremendous business value (Nambisan et al., 2017; Goyal et al., 2020). Specifically, user participation in community activities can help firms improve brand equity (Habibi et al., 2016; Rubio et al., 2020), word of mouth (Munnukka et al., 2015), and sales performance (Ho, 2015). In addition, user participation plays an important role in value co-creation: in the idea generation phase, users can provide creative and novel ideas; in the R&D phase, users can solve specific problems as a complement to the original knowledge; and in the idea commercialization phase, users can become the recipients and evangelists of the product (Poetz and Schreier, 2012; Martínez-Cañas et al., 2016). Finally, user participation in online communities has also been found to generate some negative complaints and grievances about the product and brand, which hurt word of mouth but have a positive impact in the form of improving the product or service (Hur et al., 2011).

From a broad perspective, all user behaviors in online communities can be regarded as user participation, such as browsing, replying, rating, and interacting (Zhou et al., 2014). From a narrower perspective, user participation in online communities refers to the situation in which users participate in community activities (Wang et al., 2012). The main focus of academic research has been on the motivational drivers of user participation in online communities (Liao et al., 2017; Zhou, 2020). The driving factors of user participation that have been addressed in previous studies can be grouped into three dimensions: individual factors, social factors, and information factors (Hook et al., 2018). Individual factors mainly focus on how the users themselves perceive their relationship with the community and the benefits that user participation can bring to them, specifically individual identity (Marzocchi et al., 2013), attitude (Bagozzi and Dholakia, 2006), and self-discovery (Dholakia et al., 2004). Social factors involve the interpersonal relationships formed in online communities and social benefits that community members can obtain through social interactions, such as altruism (Azar et al., 2016), social needs (Wang et al., 2012), trust (Casaló et al., 2008), and norm of reciprocity (Liao et al., 2020). Information factors primarily consider users’ participation in online communities as a way to obtain useful information. The studies in this area mainly address information needs (Wang et al., 2015) and information quality (Zhang et al., 2015). Liao et al. (2021) divided users’ idea contributions into two dimensions: quantity and quality and demonstrated that peer feedback and sponsoring firm feedback positively affect the quantity and quality of users’ ideas, social learning can increase the number of user’s ideas but decrease idea quality. In addition, they also found that direct mastery experiences have no significant effect on idea quantity but negatively affect idea quality.

### Cognitive and affective processes

The cognitive process refers to the processes through which individuals process information, including recognizing things, acquiring knowledge, and analyzing information. In contrast, the affective process describes the relationship between objective things and the subject’s needs; it provides specific information about the value judgments of things (Lemerise and Arsenio, 2000). It should be noted that cognitive processes and affective processes do not exist independently; they are reciprocal: affective processes provide specific information about the value judgments of things, and this information influences individuals’ attitudes and thinking styles. The results obtained by individuals after cognitive information processing also have an important impact on their affective evaluations (Fenske et al., 2004). In this context, Mischel and Shoda (1995) propose cognitive-affective personality system (CAPS) theory, in which they argue that people are complexes that combine rationality and sensibility and that they do not react passively to the environment but instead actively and systematically implement self-change responses. The interventions of the external environment stimulate the behavioral responses of “rational cognitive” and “emotional impulses,” ultimately determining the behavioral choices of individuals (Mendoza-Denton et al., 1997).

A large number of studies based on the CAPS theoretical framework have explored the influence of both cognitive and affective simulation on consumer behavior. Ahn and Back (2020) find that customers’ affective and cognitive elaborations are elicited concurrently in the formation of brand relationship quality and behavioral intention. Regarding the impact mechanism of cognitive and affective information on consumer behavior, studies have demonstrated that consumer engagement can be the mediator, which contains cognitive-level engagement and affective-level engagement (Dessart, 2017; Rather and Hollebeek, 2021). In addition, individual differences, such as the need for cognition (NFC) and need for affect (NFA), have been found to play a significant moderating role in the influence of cognitive and affective information on individuals’ behavioral decisions. Specifically, affective information triggers more positive attitudes in individuals with high NFA and low NFC, while cognitive information triggers more positive attitudes in individuals with low NFA and high NFC (Haddock et al., 2008).

### Mental simulation theory

Taylor and Schneider (1989) first proposed the concept of mental simulation, defining it as an individual’s simulated mental representation of an event or series of events. In the field of information systems and marketing, scholars have focused on the anticipatory nature of mental simulation and have defined it as the simulated imagination of consumers about (unused or experienced) product interactions (Hoeffler, 2003). Such simulated imagery can increase the perceived realism and validity of imagined content for consumers at the cognitive level, contributing to individuals’ behavioral decisions (Elder and Krishna, 2012). Moreover, mental simulation can affect individuals not only at the cognitive level, but it may also be accompanied by a strong affective response, which can effectively evoke behavioral motivation in consumers (Taylor et al., 1998). Zhao et al. (2014) have demonstrated that consumers who start mental simulation are more likely to act out the imagery and buy the product. Therefore, in practice, mental simulation theory is a widely used marketing strategy in advertising or product promotion, (Zhao et al., 2014).

Mental simulation can be divided into process simulation and outcome simulation. Process simulations guide customers in imagining the process of using a product or the action steps they must take to obtain a new product, while outcome simulations guide customers in imagining the results and benefits of using or obtaining a product (Sun et al., 2018). Previous studies have found that process simulation and outcome simulation have different influence mechanisms on individual behavior: process simulations help people visualize the steps and processes required to achieve their goals, and outcome simulations allow people to visualize the results and benefits of achieving those goals (Pham and Taylor, 1999). In consumers’ new product adoption decisions, outcome simulations reduce new product uncertainty and psychological costs, while process simulations reinforce positive emotions, thus increasing behavioral intentions (Castaño et al., 2008). Zhao et al. (2011) investigate the role of process simulation and outcome simulation in consumer product evaluations under cognitive and affective information processing. Results indicate that outcome simulation is more effective than process simulation in increasing product evaluation under a cognitive mode, whereas process simulation is more effective than outcome simulation under an affective mode. They also find the effect would reverse for hedonic products and the distant future.

User participation behavior is the key to the operation and development of online communities and is of great value to the development, improvement, and promotion of new products. Prior studies have focused on the antecedents and consequences of user participation behavior in online communities (Hook et al., 2018); however, there is a lack of studies considering the guidance of user participation behavior, especially at the linguistic level. The cognitive and affective processes are two important paths for users in their processing of information, but the roles and impact mechanisms of both paths on user participation in online community activities are still unclear. To fill this gap, the current study has explored the impact and underlying mechanism of cognitive and affective guidance on user participation in online innovation activities. Based on existing theories and literature, we also examined the mediating effect of mental simulation and moderating effect of product types. We have referred to Zhao et al. (2011) on the impact of cognitive and affective factors on product evaluation; however, it should be noted that there are also some differences between online community participation and product evaluation. First, in product evaluation, there is information asymmetry, in which consumers may feel high uncertainty, whereas, in participation behavior, users share their suggestions based on their experiences, in which consumers will face less uncertainty. Second, in product evaluation, users spend more effort because misjudgment can lead to mistakes in subsequent behaviors, such as purchasing or recommending. Therefore, existing research conclusions cannot fully explain the research questions raised in the current study, indicating that more research is needed to explore the impact of innovation guidance on user participation behavior in online communities.

## Hypothesis development

### Innovation guidance and user participation

At the beginning of innovation activities, companies typically present users with a paragraph of guidance text that introduces the community activities and attracts them to participate in the activities. According to stimulus-organism-response (SOR) theory (Jacoby, 2002), innovation guidance can be regarded as an external simulation, and users influenced by the information will unconsciously generate images containing the product and even simulate consumption experiences. This imagination or simulation actively impacts users, making them eager to share their feelings and experiences about the product (Zhao et al., 2009). Thus, we argue that innovation guidance can significantly increase user participation in online innovation activities. In terms of guidance methods, online communities usually adopt cognitive guidance and affective guidance. Cognitive guidance provides product information, such as features and quality, which can help users target products of interest quickly. In addition, cognitive guidance can also provide a stronger direction in helping users express themselves more clearly and participate in activities more effectively (Li and Huang, 2020). Affective guidance is another effective way to motivate users to participate in online community innovation activities. Previous studies have shown that users are more likely to engage in activities containing positive emotions and are more willing to communicate with positive people and share positive information (Mehdizadeh, 2010). Considering that almost all affective guidance in online community activities is positive, participating in the activities not only makes users happy, but it also helps them create a positive social image; this self-image shaping will motivate them to participate more actively in activities. Therefore, we hypothesize the following:

*H*1: Innovation guidance can significantly boost user participation in online innovation activities.

*H*1a: Cognitive guidance can facilitate user participation in online innovation activities.

*H*1b: Affective guidance can facilitate user participation in online innovation activities.

### The mediating effect of outcome simulation

Outcome simulation focuses on the desired outcomes and benefits of using a specific product (Rather and Hollebeek, 2021). When confronted with a product of interest, innovation activity guidance can encourage users to generate imagery related to product features or use (Feiereisen et al., 2013). When users are confronted with quality information and functional information about a product, they will unconsciously imagine the convenience they will have when using the product. For example, when users are exposed to the related information an iRobot, such as the functions of sweeping, mopping, and automatic cleaning, they will imagine that using this product will help them reduce the housework burden. Compared with affective guidance, cognitive guidance consists of more information about the features and functions of products, and this kind of information is more likely to evoke the user’s imagination about the outcome after utilizing the function, causing the user to initiate the outcome simulation.

Imagery generated by users about the results of product use can be viewed as a cognitive construction created by the users themselves, which will lead them to seriously consider the benefits the product brings to their lives (Escalas, 2004). This perception of convenience helps improve their overall evaluation and perception of the value of the product (Voss et al., 2003). When users perceive a stronger product value, they are more likely to engage in online community activities and share their creativity because of factors such as reciprocity and self-image. Building on this, we hypothesize the following:

*H*2a: Innovation guidance can evoke users to perform an outcome simulation, and cognitive guidance is more likely to facilitate users in initiating an outcome simulation than affective guidance.

*H*2b: The outcome simulation generated by innovation guidance positively influences user participation.

### The mediating effect of process simulation

Process simulation focuses on a person’s ability to imagine or recall what is necessary to accomplish a task (Voss et al., 2003). Guidance in online community activities generally constructs usage scenarios when conveying product information. After browsing this product information, users will recall their previous experience with the product (Kisielius and Sternthal, 1984). For example, when users read the description that a projector can bring them a private theater-like visual and auditory feast, they are easily brought into the scenario and imagine enjoying their leisure time with friends or family. Compared with cognitive guidance, affective guidance includes more situational language and positive emotions; this type of information is more likely to evoke the user’s imagination of the process of using the product, making users initiate the process simulation.

By imagining the process of using the product, users can enhance the richness of the information they have, helping increase the users’ information-sharing intention (Zhou et al., 2021). On the other hand, the product use process is often accompanied by users’ emotional experiences, and positive emotional experiences are considered to be the key to arousing user behavior (Carroll, 1978). When users obtain positive emotions in a process simulation, their information-sharing behaviors and online community activity participation will be enhanced. Thus, we hypothesize the following:

*H*3a: Innovation guidance can encourage users to engage in process simulation, and affective guidance is more likely to facilitate users to initiate in process simulation than cognitive guidance.

*H*3b: The process simulation generated by innovation guidance positively influences user participation.

### The moderating effect of product type

The literature has indicated that consumers have different attitudes toward hedonic products and utilitarian products, and this attitude difference will lead to a difference in their behavior (Lim and Ang, 2008). In the current study, the difference between hedonic products and utilitarian products is reflected in the user’s emotional state being evoked: hedonic products are pleasure oriented, and consumption is mainly caused by the desire for fun, experience, and enjoyment. Products with this property allow consumers to experience a sense of freedom and pleasure. In contrast, utilitarian products match more strongly with consumers’ rational appeals and allow consumers to experience a sense of convenience and durability (Okada, 2005). In community innovation activities, cognitive guidance places more emphasis on product features and functions. This information can better meet consumers’ needs for utilitarian products, which can help consumers evaluate products more comprehensively and make faster judgments. Therefore, utilitarian products can be better integrated with cognitive guidance. Affective guidance places greater emphasis on elaborating on the fun and enjoyment brought by the product, which is consistent with the pleasure-oriented nature of hedonic products. Therefore, hedonic products are more suitable for affective guidance. We conclude that to achieve better motivational effects on user participation, it is necessary to adopt matching guidance methods for different product types in community innovation activities.

An outcome simulation enables users to pay more attention to the actual effects of the product, such as cost-effectiveness and functionality (Okada, 2005). Considering utilitarian products are concerned with functional attributes, when faced with cognitive guidance, users tend to pay more attention to the quality, functionality, and practical effects of the product during the outcome simulation. Process simulation emphasizes the pursuit of pleasurable feelings, which leads consumers to pay more attention to attributes such as entertainment and experience (Okada, 2005). Because hedonic products pursue the attributes of experience and sensory pleasure, when faced with affective guidance, users will amplify the pleasure brought about by the products and enjoy the process of using them during the process simulation. Therefore, we suggest that product type can moderate the effect of innovation guidance on outcome simulation and process simulation. In addition, we also expect the matching effect will occur between product types and innovation guidance methods. Specifically, the match between cognitive guidance (affective guidance) and utilitarian products (hedonic products) leads to greater outcome simulation and process simulation. Therefore, we propose the following hypothesis:

*H*4a: Product type moderates the effect of innovation guidance on outcome simulation. For utilitarian products (hedonic products), cognitive guidance (affective guidance) can lead to greater outcome simulation.

*H*4b: Product type moderates the effect of innovation guidance on process simulation. For utilitarian products (hedonic products), cognitive guidance (affective guidance) can lead to greater process simulation.

**Figure 1:**
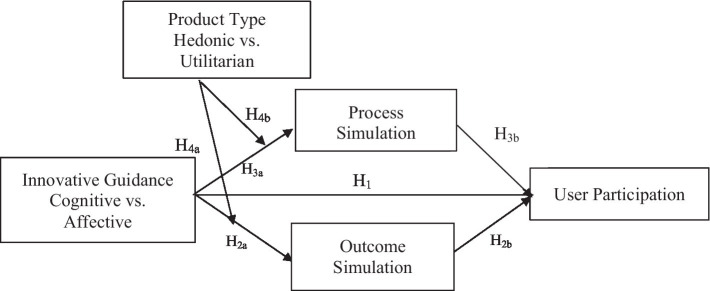
The conceptual model.

We present a conceptual model of the impact mechanism of innovation guidance (cognitive guidance vs. affective guidance) on user participation behavior in online communities below (as shown in Figure 1) before combining econometric analysis and experimental research to verify this conceptual model through three substudies.

## Study 1

### Data source

We first examined the effects of cognitive guidance and affective guidance on online community participation behaviors, here based on secondary data from online platforms. The data were collected from NetEase’s Strict Selection community. NetEase’s Strict Selection is an e-commerce platform for home furnishing and daily necessities. Since its establishment in 2016, it has become a leading B2C shopping website in China. It should be noted that NetEase’s Strict Selection also has an online community, which plays an important role in product development and promotion. In this online community, the platform will periodically launch opinion-gathering activities, which include a paragraph of guidance text, to familiarize users with the purpose of the activity and motivate them to participate. Users are asked to suggest their ideas for the product’s development within a certain time. We collected data from 216 opinion-gathering activities with specific data, including product names, the text of activity guidance, and the number of users participating in each event.

### Variable description

The dependent variable was user participation, which was measured by the number of users posting to each activity topic. The independent variables included cognitive guidance and affective guidance. We extracted the linguistic features from the guidance text using the Linguistic Inquiry and Word Count (LIWC) program. LIWC was developed by Pennebaker et al. (2003) for word frequency analysis as a way to measure the number of words in a given text that reflect particular linguistic or psychological processes and spoken language categories. We used the proportion of cognitive words and affective words to represent cognitive guidance and affective guidance, respectively. We also created a dummy variable for product type and set it to 0 if the product belonged to utilitarian products and 1 for hedonic products. The product types were distinguished mainly by three research assistants, who provided manual coding work based on the definition of product types. The results of three research assistants’ classifications were highly consistent, ensuring the accuracy of the coding.

To acquire a valid estimate, some product-based features should be considered control variables. First, different products may receive different degrees of user attention, which can affect users’ participation behavior. We used the Baidu search index to measure product attention. The Baidu search index contains the statistics for the volume of Internet searches for specific keywords at a given time, which is widely used to measure user attention (Jin et al., 2015). In the current study, we counted the Baidu search indexes of the subject products during the opinion-gathering activities and calculated their daily average values. Second, we included the price to control the difference in users’ preferences for products with different values. Third, we also included the variable of length to control the influence of the length of the guidance text. In Table 1, we describe the variables and summarize the descriptive statistics of the key variables.

**Table 1:** Variables, measures, and descriptive statistics.

Variables	Measures	Mean	SD	Max.	Min.
User participation	Number of users posting to each activity topic	120.324	83.815	388.000	18.000
CogGuidance	The proportion of cognitive words	0.170	0.055	0.308	0.000
AffGuidance	The proportion of affective words	0.051	0.028	0.125	0.000
Product type	Dummy variable, 0 for utilitarian products and 1 for hedonic products	0.412	0.493	1.000	0.000
Price	Product price	291.731	845.429	10825.000	9.900
Attention	The average value of the product Baidu search index during the activity period	778.213	1054.744	8494.000	7.000
Length	Number of words in the guidance text	50.324	11.639	69.000	19.000

### Results

Considering that the dependent variable belonged to count data and the variance of the dependent variable differed significantly from the mean value, a negative binary regression was applied to our study (Jin et al., 2015). A negative binary regression analysis was conducted using Stata14; the results are shown in Table 2.

**Table 2:** Regression results.

Variables	Model 1	Model 2	Model 3	Model 4	Model 5	Model 6	Model 7
CogGuidance	—	0.061*** (0.011)	0.076*** (0.011)	0.064*** (0.011)	−0.011 (0.028)	0.086*** (0.017)	0.064*** (0.011)
AffGuidance	—	0.017** (0.006)	0.016*** (0.006)	−0.007 (0.012)	0.031*** (0.006)	−0.008 (0.012)	0.022*** (0.006)
Length	−0.161*** (0.036)	−0.217*** (0.034)	−0.207*** (0.034)	−0.236*** (0.034)	−0.148*** (0.047)	−0.300*** (0.054)	−0.223*** (0.034)
Price	−0.015* (0.009)	−0.017** (0.009)	−0.017* (0.009)	−0.017** (0.008)	−0.021** (0.010)	−0.014 (0.013)	−0.017* (0.009)
Attention	0.041*** (0.008)	0.035*** (0.008)	0.037*** (0.008)	0.034*** (0.008)	0.038*** (0.011)	0.035*** (0.010)	0.037*** (0.008)
Product type	0.058*** (0.022)	0.065*** (0.021)	−0.008 (0.061)	0.209*** (0.050)	—	—	0.063*** (0.021)
CogGuidance *Product Type	—	—	−0.077*** (0.029)	—	—	—	—
AffGuidance * Product Type	—	—	—	0.044*** (0.013)	—	—	—
CogGuidance * AffGuidance	—	—	—	—	—	—	−0.047*** (0.011)
Constant	2.368*** (0.165)	2.368*** (0.165)	2.336*** (0.170)	2.370*** (0.164)	2.073*** (0.265)	2.631*** (0.250)	2.400*** (0.164)
Observations	216	216	216	216	89	127	216

* *p* < 0.1;

** *p* < 0.05;

*** *p* < 0.01.

Model 1 (Column 2 in Table 2) presents our baseline model, indicating the effect of control variables on the dependent variable user participation. The length of the guidance text was negatively associated with user participation (−0.161, *p* < 0.01). In studies on online reviews, conclusions about the impact of length on the perceived usefulness of consumers have been inconsistent: some studies argue that longer online reviews are more effective at attracting users’ attention (Kuan et al., 2015) and mitigate product-related uncertainty (Hu and Chen, 2016). Some studies have indicated that long online reviews can cause cognitive overload on users, so there may be an inverted U-shaped relationship between length and the perceived usefulness of consumers (Fink et al., 2018). Our study has supported the latter view, suggesting that more concise and accurate expressions should be used to guide innovation activities.

We first examined the main effects of cognitive guidance and affective guidance on user participation. We report the results in Model 2 (Column 3 in Table 2), which shows a significant and positive impact of cognitive guidance on user participation (0.061, *p* < 0.01), supporting hypothesis H1a. In addition, affective guidance also had a significant and positive impact on user participation (0.017, *p* < 0.05), thus supporting hypothesis H1b. To examine the moderating effect of product type, we constructed interaction terms for cognitive guidance and product type, and affective guidance and product type, respectively. The results presented in Model 3 (Column 4 in Table 2) and Model 4 (Column 5 in Table 2) show that the interaction between cognitive guidance and product type is significant and negative (−0.077, *p* < 0.01), whereas the interaction between affective guidance and product type is significant and positive (0.044, *p* < 0.01). This result indicates that cognitive guidance is more effective for utilitarian products, and affective guidance is more effective for hedonic products. To further compare the difference between the effects of cognitive guidance and affective guidance under different product types, we split the dataset into two subgroups according to the product types and ran the regression program. Model 5 (Column 6 in Table 2) presents the results for hedonic products, and Model 6 (Column 7 in Table 2) demonstrates the results for utilitarian products. For hedonic products, affective guidance positively influenced user participation (0.031, *p* < 0.01), and the impact of cognitive guidance was not significant (−0.011, *p* > 0.05). Conversely, for utilitarian products, cognitive guidance positively influenced user participation (0.086, *p* < 0.01), and the impact of affective guidance was not significant (−0.008, *p* > 0.05). In practice, the relationship between cognitive guidance and affective guidance may not be completely independent. Some innovation guidance texts may contain both cognitive and affective components. To explore the effect of a mixed guidance method, we constructed the interaction terms of cognitive guidance and affective guidance, adding them to our regression model. The results presented in Model 7 (Column 8 of Table 2) show that the interaction term is negative and significant (−0.047, *p* < 0.01), indicating that cognitive guidance (affective guidance) weakened the effect of the original affective guidance (cognitive guidance) on user participation.

## Study 2

### Pre-experiment

The pre-experiment aimed to verify the effectiveness and variability of the manipulation of cognitive guidance and affective guidance. For the experimental product, we chose a table lamp because it is a neutral product with both hedonic and utilitarian properties. To determine the cognitive and affective guidance texts, as references, we selected five activities from Study 1 that had the highest cognitive and affective components and edited the content to make sure it matched the experimental product. The pre-experiment invited 42 undergraduates and postgraduates from a university in China. The participants were randomly divided into three groups (cognitive group, affective group, and control group). First, we introduced the concepts of cognitive guidance and affective guidance to the participants before providing them with the guidance text. Cognitive guidance was a merchant/platform that could help users know more about the product by displaying information about the product’s attributes and other information. Affective guidance was the merchant’s/platform uses contextualized context and emotional language to describe the product and arousal user’s emotion. After reading the guidance texts of the innovation activity, they were asked to report the cognitive and affective components they felt. The measurement used a 7-point Likert scale, with 1 being “very weak,” and 7 being “very strong.” In addition, we asked the participants to provide their demographic information, such as gender and age.

For gender, there were no significant differences in the perceptions of cognitive and affective components between males (40.5%) and females (59.5%), so we pooled data from men and women for analysis. The cognitive group reported a stronger cognitive component than the control group (*M* = 6.21, *SD* = 0.58 vs. *M* = 3.00, *SD* = 0.88, *t*(13) = 11.44, *p* < 0.05); the affective group reported a stronger affective component than the control group (*M* = 6.29, SD = 0.73 vs. *M* = 2.79, *SD* = 0.80, *t*(13) = 12.11, *p* < 0.05). Therefore, the pre-experiment was successful in manipulating both cognitive and affective guidance. The guidance text was deemed acceptable for use in a formal experiment.

### Experimental design and measures

Study 2 aimed to test the dual mediating effects of the two types of mental simulation—process simulation, and outcome simulation—in the innovation guidance method on user participation behavior. Here, 151 participants (79 males/72 females) were recruited from a university in China. The participants were randomly assigned to three groups: the cognitive group, the affective group, and the control group. Specifically, 54 participants were assigned to the cognitive group, 46 participants were assigned to the affective group, and 51 participants were assigned to the control group. At the beginning of the study, we informed the participants that they needed to report their participation intentions after reading the guidance given by the innovation activity. Specifically, we told them to imagine that they were users in the online community. Then, we provided the participants in the cognitive group and affective group with the cognitive guidance text and affective guidance text, respectively. The guidance text provided to the control group did not contain any cognitive or affective components. After reading the guidance text, the participants were asked to use the scales on the questionnaire to report the process simulation, outcome simulation, and participation intention.

The process simulation and outcome simulation were measured on the three-item and two-item scales developed by Escalas and Luce (2004). Cronbach’s alpha was 0.822 and 0.817, respectively. User participation was measured on the three-item scales developed by Lin (2006). Cronbach’s alpha was 0.866.

### Experimental results

First, we examined the effects of cognitive guidance and affective guidance on user participation. The results of the independent sample *t*-test indicated that user participation was significantly higher in the cognitive group than in the control group (*M* = 5.86, *SD* = 0.89 vs. *M* = 4.13, *SD* = 0.76, *t*(103) = 10.65, *p* < 0.05), so hypothesis H1a was supported. User participation behavior was significantly higher in the affective group than in the control group (*M* = 5.54, *SD* = 1.18 vs. *M* = 4.13, *SD* = 0.76, *t*(95) = 7.05, *p* < 0.05), so hypothesis H1b was supported. We also compared user participation between the cognitive group and affective group, and the results showed that there was no significant difference in user participation behavior between the cognitive and affective groups (*M* = 5.86, *SD* = 0.89 vs. *M* = 5.54, SD = 1.18, *t*(98) = 1.55, *p* = 1.25 > 0.05).

Next, we compared the effect of cognitive guidance and affective guidance on mental simulation. We found that the outcome simulation in the cognitive group was significant and stronger in the affective group (*M* = 5.86, *SD* = 0.93 vs. *M* = 5.35, *SD* = 0.98, *t*(98) = 2.68, *p* < 0.05), supporting hypothesis H2b. For the process simulation, we drew the opposite conclusion: the process simulation in the affective groups was significant and stronger in the cognitive group (*M* = 5.62, *SD* = 0.86, *t*(98) = 2.58, *p* < 0.05), hence supporting hypothesis H3b.

Finally, we explored the role of outcome simulation and process simulation on the impact of innovation guidance methods on user participation. To test the effects of outcome simulation and process simulation on user participation, we set user participation as the dependent variable and process simulation and outcome simulation as independent variables and then conducted regression analysis. The results showed that both outcome simulation (*β* = 0.24, *p* < 0.05) and process simulation (*β* = 0.21, *p* < 0.05) significantly and positively affected user participation. Then, we coded cognitive guidance as 1 and affective guidance as 0 and tested the dual mediating effect. Following Zhao et al. (2010) and Hayes (2017), we used the conditional process analysis program PROCESS, which can compute ordinary least square regressions to test for direct and indirect effects. We employed PROCESS Model 4 to estimate the regression coefficients and follow-up bootstrap analyses with 5,000 bootstrap samples to estimate the 95% bias-corrected confidence intervals for specific and total indirect effects. The results showed that both outcome simulations and process simulations mediated the effect of the innovation guidance method on user participation behavior, here with mediated effect sizes of 0. 113 (LLCI = 0.002, ULCI = 0.27) and −0.257 (LLCI = −0.47, ULCI = −0.06), so the mediating effect hypotheses for Hypothesis H2a and Hypothesis H3a were also validated.

## Study 3

### Pre-experiment

The purpose of the pre-experiment was to determine the experimental product, ensure the validity of the formal experimental manipulation, and avoid any deviation of the experimental results caused by improper product selection. Referring to the research (Arifine et al., 2019), we selected chocolates, fruit juice, and milk tea as options for hedonic products, and shampoo, umbrellas, and milk as options for utilitarian products. The pre-experiment recruited 42 undergraduates and postgraduates from a university in China. First, the subjects were shown definitions of utilitarian products and hedonic products. Then, the subjects were asked to report their perceived utility degree and hedonic degree of six products using a 7-point Likert scale, with 1 being very weak and 7 being very strong. Finally, shampoo was identified as a functional product and chocolate as a hedonic product.

### Experimental design

The purpose of Study 3’s formal experiment was to examine the moderating effects of product type. A total of 318 subjects (159 males and 159 females) from a university in China participated for monetary incentives. They were randomly assigned to one of four conditions of a 2 (innovation guidance method: cognitive guidance vs. affective guidance) × 2 (product type: utilitarian products vs. hedonic products) between-subjects factorial design. Specifically, 78 participants were assigned to the cognitive guidance and utilitarian products condition, 82 to the cognitive guidance and hedonic products condition, 80 to the affective guidance and utilitarian products condition, and 78 to the affective guidance and hedonic products condition. The procedure was the same as in Study 2. Each group of participants explained the purpose of the innovation activity and was provided with the activity guidance text. After reading the guide, they were asked to complete the scales of process simulation, outcome simulation, and user participation. In addition, we asked the participants to report the cognitive and affective components they felt from the guidance text and hedonic and utilitarian components they felt about the product. We used the same scales as in Study 2. The Cronbach’s alpha of process simulation, outcome simulation, and user participation were 0.839, 0.853, and 0.814, respectively, indicating that the scales had good reliability.

### Experimental results

For the manipulation check of product types, the hedonic group reported a stronger perceived hedonic component than the utilitarian group (*M* = 5.65, *SD* = 0.95 vs. *M* = 2.37, *SD* = 0.95, *t*(40) = 24.06, *p* < 0.05); the utilitarian group reported a stronger perceived utilitarian component than the hedonic group (*M* = 5.80, *SD* = 1.22 vs. *M* = 2.30, *SD* = 0.90, *t*(280) = 27.62, *p* < 0.05). For the manipulation check of guidance methods, the cognitive group reported a stronger perceived cognitive component than the affective group (*M* = 5.93, *SD* = 0.83 vs. *M* = 1.82, *SD* = 0.66, *t*(306) = 48.07, *p* < 0.05); the affective group reported a stronger perceived affective component than the cognitive group (*M* = 5.58, *SD* = 1.05 vs. *M* = 2.31, *SD* = 0.88, *t*(306) = 29.81, *p* < 0.05). Hence, our manipulations of product type and guidance methods ere successful.

First, we examined the moderating effect of product type on the impact of innovation guidance methods on mental simulation. In the current study, cognitive guidance and affective guidance were coded as 0 and 1, respectively; utilitarian and hedonic products were coded as 0 and 1, respectively. Referring to Yi et al. (2017), multivariate analysis of variance (MANOVA) was first conducted on both outcome simulation and process simulation. Pillai’s trace test revealed a significant interaction effect between innovation guidance methods and product type (*p* < 0:05). We then used follow-up ANOVAs to test the effects on the two dependent variables outcome simulation and process simulation separately.

The cell statistics and ANOVA test are shown in Tables 3, 4. ANOVA Results on outcome simulation showed a significant two-way interaction effect between innovation guidance methods and product type (*F*(1, 304) = 5.05, *p* < 0.05). The main effect of innovation guidance methods (F(1, 304) = 54.72, *p* < 0.05) and product type (F(1, 304) = 4.47, *p* < 0.05) was also significant. For the process simulation, the interaction effect of innovation guidance methods and product type was also significant (*F*(1, 304) = 6.11, *p* < 0.05), while the main effect of innovation guidance method (*F*(1, 304) = 41.31, *p* < 0.05) and product type (*F*(1, 304) = 10.04, *p* < 0.05) was significant.

**Table 3:** Means and standard deviations of the four conditions.

	Utilitarian products	Hedonic products
*Outcome simulation*		
Cognitive guidance	5.87 (0.61)	5.42 (0.73)
Affective guidance	4.78 (1.00)	4.88 (1.21)
*Process simulation*		
Cognitive guidance	4.69 (0.90)	4.76 (1.04)
Affective guidance	5.10 (0.92)	5.68(0.77)

**Table 4:** ANOVA test—main and interaction effects.

	Dependent variable	*df*	Mean Square	*F*	Sig.
Innovation guidance	Outcome simulation	1	44.76	54.72	0.00
	Process simulation	1	33.61	41.31	0.00
Product type	Outcome simulation	1	3.66	4.47	0.04
	Process simulation	1	8.17	10.04	0.02
Innovation guidance*Product type	Outcome simulation	1	4.13	5.05	0.03
	Process simulation	1	4.97	6.11	0.01

**Figure 2:**
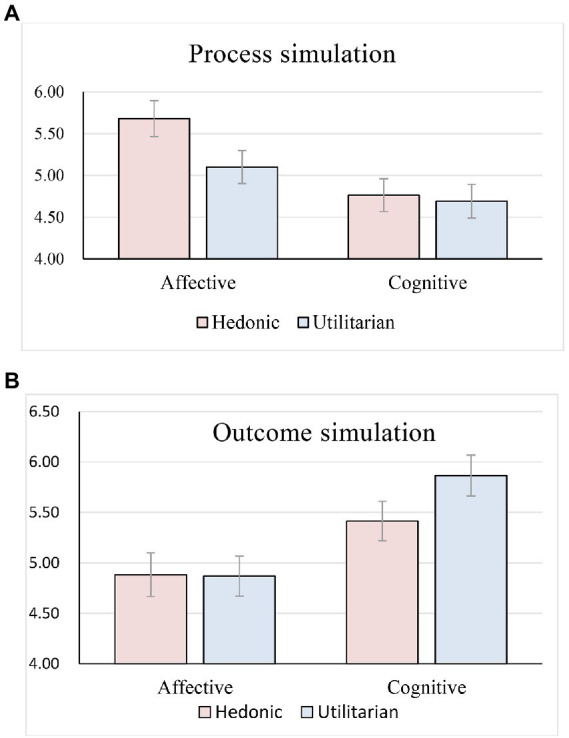
**(A)** Simple mean effect analysis of process simulation. **(B)** Simple mean effect analysis of outcome simulation.

To further explore the moderating effect, we conducted a simple mean effect analysis. Simple effect analysis confirmed that affective guidance is more effective than cognitive guidance to evoke process simulation for hedonic products (*p* < 0:05). However, for utilitarian products, there is no significant difference between the two innovation guidance methods in promoting process simulation (*p* > 0.05; see Figure 2A), thus hypothesis H4a was partially supported. In addition, a simple effect analysis of outcome simulation showed that cognitive guidance is more effective than affective guidance to evoke outcome simulation for utilitarian products (*p* < 0:05). For utilitarian products, there is no significant difference between the two innovation guidance methods in promoting process simulation (*p* > 0.05; see Figure 2B), hypotheses H4b was also partially supported.

Finally, we performed moderated mediation tests using the regression bootstrapping method in the PROCESS module (Model 7), as developed by Hayes (2017). Here, the effect of guidance methods on user participation *via* outcome simulation was positive and significant for both hedonic products (0.32, LLCI = 0.20, ULCI = 0.48) and utilitarian products (0.17, LLCI = 0.07, ULCI = 0.28), and the confidence interval for the difference between these two effects did not cross zero. The effect of the guidance methods on user participation *via* process simulation was negative and significant for both hedonic products (−0.35, LLCI = −0.52, ULCI = −0.20) and utilitarian products (−0.15, LLCI = −0.27, ULCI = −0.05); the confidence interval for the difference between these two effects did not cross zero.

## Discussion

### Conclusion

In the current study, we investigated the impact of cognitive guidance and affective guidance on user participation and the underlying mechanism through secondary data analysis and two experiments. We can draw the following key findings: First, both cognitive guidance and affective guidance can facilitate user participation in online innovation activities. Previous research has examined the effects of cognitive and affective information on individual attitudes (Haddock et al., 2008) and behavioral intention (Ahn and Back, 2020), this conclusion extends this research by validating the motivational role of cognitive and affective information in user participation. Second, outcome simulations and process simulations play a dual mediating role in the impact of innovation guidance on user participation. Moreover, we also found that cognitive guidance (affective guidance) is more likely to facilitate users’ initiation of outcome simulation (process simulation) than effective guidance (cognitive guidance). Third, product type moderates the dual mediating effect of innovation guidance on user participation *via* outcome simulations and process simulations. We also found that affective guidance is more motivating in process simulation for hedonic products, while cognitive guidance is more motivating in outcome simulation for utilitarian products. These findings are inconsistent with Zhao et al. (2011). The reason may due to the different stability of individual product preferences. As Zhao et al. (2011) proposed that when evaluating products, the preferences of users are typically not well-formed. However, users of online communities usually have some experience using the product and their product preferences are well-formed.

### Theoretical contribution

To the best of our knowledge, the present study is the first mixed methods investigation that explores the effect of innovation guidance on user participation at both the activity level and the individual level. Our study contributes to theory and research in several ways. First, the literature on user involvement in innovation in online communities has mostly focused on user participation motivation and its influencing factors, which means there is a relative lack of research considering innovation guidance issues (Hook et al., 2018). By combining secondary data analysis and experimental methods, the current study sheds light on the role of cognitive guidance and affective guidance in online innovation activities, enriching the theoretical understanding of user participation incentives. Second, although the drivers of user participation have been examined considerably, the underlying mechanism has rarely been investigated (Liao et al., 2020). Based on mental simulation theory, the present study has further investigated the underlying mechanism of the influence of innovation guidance methods on user participation, verifying the dual mediating role of process simulation and outcome simulation. This result expands the scope of mental simulation theory and provides a new perspective for online community research. Third, we have further explored the boundary conditions of the impact of innovation guidance in online communities. Wang et al. (2013) find that utility needs and hedonistic needs are the main drivers behind user interactions. Our research enriches this theory of user behavior by testing the matching effect between innovation guidance methods and product types, which also provides a new direction for future research.

### Practical implications

The findings of our study also have significant implications for practice. First, enterprises or platforms should adopt appropriate guidance methods according to the product type in online innovation activities. For hedonic products, enterprises or platforms should use more affective expressions and contextual content in their activity guidance. For utilitarian products, more cognitive expressions and product features should be used in the activity guidance to help users associate the product with its intended effects. Second, in online innovation activities, we recommend that enterprises or platforms use a single type of innovation guidance rather than a mixed guidance method because the mixed guidance would distract users and diminish the promotion effect of the original guidance method. In addition, the innovation guidance needs to be more concise and avoid too long words because excessively long guidance will increase the cognitive load of users, reduce their desire to read, and, thus, reduce their motivation to participate in innovation activities.

### Limitations and future research

The current study is subject to some inevitable limitations, all of which provide promising directions for future research. First, the measurement of user participation using the number of users participating in innovation depends more on the breadth of user participation in innovation; however, the depth of participation was not considered. Liao et al. (2021) used stamps to measure the quality of user contribution; however, for websites without stamps, a text mining approach can be used to measure the quality of user contribution. Second, in terms of product types, we considered only the classification of utilitarian products and hedonic products. Future research could adopt other product classification methods, such as search products and experiential products, high involvement products, and low involvement products. Third, we have mainly investigated the impact mechanism of innovation guidance methods in online communities from an individual psychological level without taking into account the possible social influences and community behaviors in the community (Dholakia et al., 2004; Ho, 2015). Future studies could further investigate this issue from new research perspectives.

## Data availability statement

The data analyzed in this study are subject to the following licenses/restrictions: the raw/processed data required to reproduce these findings cannot be shared at this time as the data also form part of an ongoing study. Requests to access these datasets should be directed to cnjnly@sdnu.edu.cn.

## Author contributions

YL contributed to all the phases of the study from conception and design of the study, statistical analysis and results interpretation. XG contributed to theoretical literature review, data collection and writing the first draft. HH contributed to conception of the study and data collection. HZ contributed to supervision and the revision of the work. All authors discussed the results and contributed to the final manuscript.

## Funding

This article was supported by Shandong Youth Innovation Technology Program (“Youth Sicence and Technology Innovation Plan” in Universities of Shandong Province, 2020RWG001), Humanities and Social Science Project of Shandong Province (2021-JCGL-05), and Shandong Social Science Planning Research Project (22CGLJ37).

## Conflict of interest

The authors declare that the research was conducted in the absence of any commercial or financial relationships that could be construed as a potential conflict of interest.

## Publisher’s note

All claims expressed in this article are solely those of the authors and do not necessarily represent those of their affiliated organizations, or those of the publisher, the editors and the reviewers. Any product that may be evaluated in this article, or claim that may be made by its manufacturer, is not guaranteed or endorsed by the publisher.
