# Supplementary material for: Exploring the impact of innovation guidance on user participation in online communities: A mixed methods investigation of cognitive and affective perspectives
Source: Front Psychol. 2022 Sep 28;13:1011837. doi: 10.3389/fpsyg.2022.1011837 (PMC9554656; doi:10.3389/fpsyg.2022.1011837)
Supplement: Supplementary file 1 [file Data_Sheet_1.docx]

**APPENDIX A. EXPERIMENT MANIPULATION FOR STUDY 2**


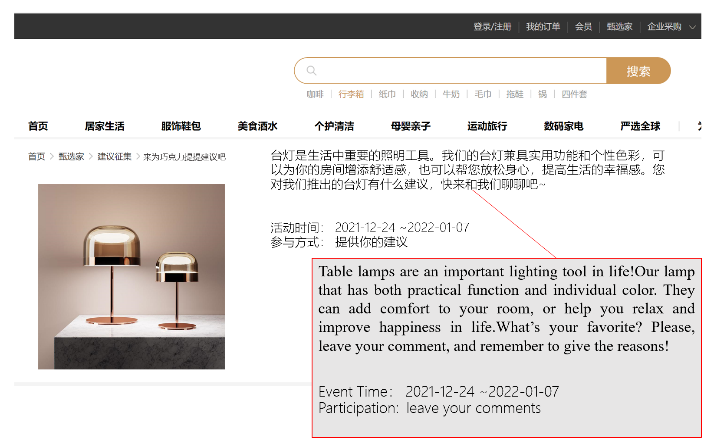


**Affective Group**


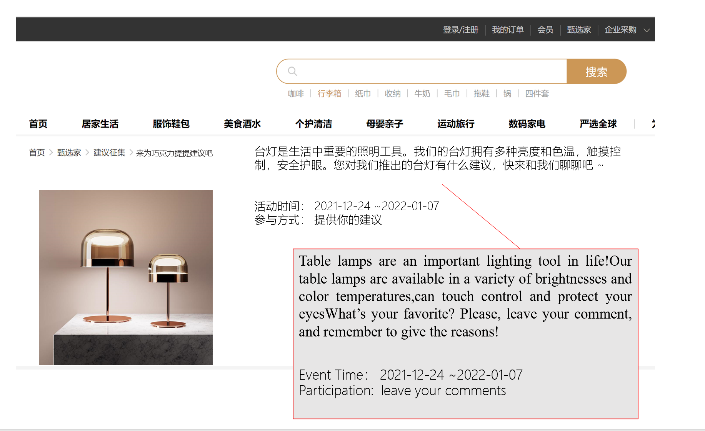


**Cognitive Group**

**Control Group**


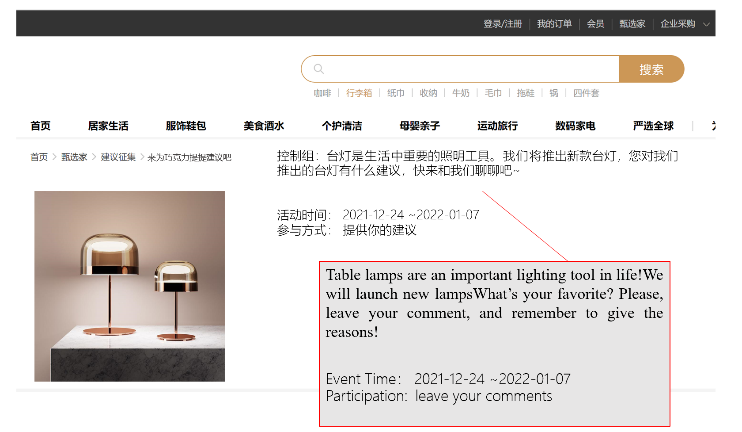


**APPENDIX B. EXPERIMENT MANIPULATION FOR STUDY 3**


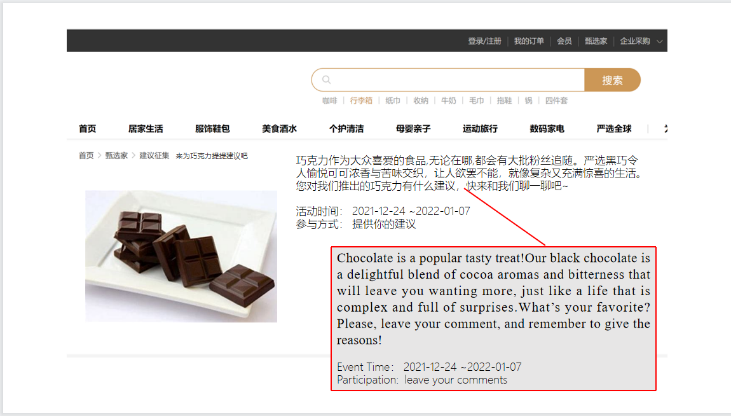


**Hedonic—Affective Group**


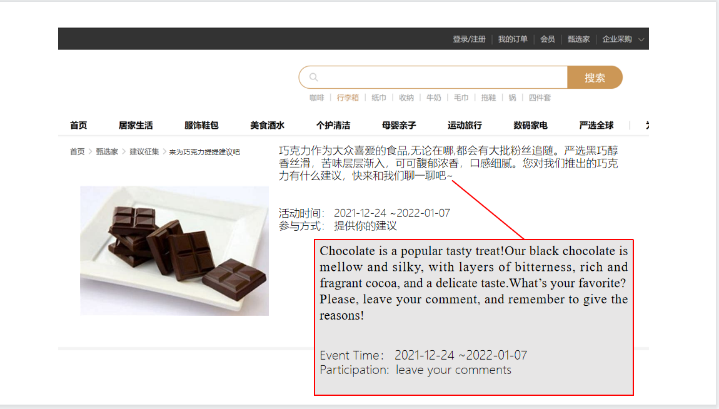


**Hedonic—Cognitive Group**


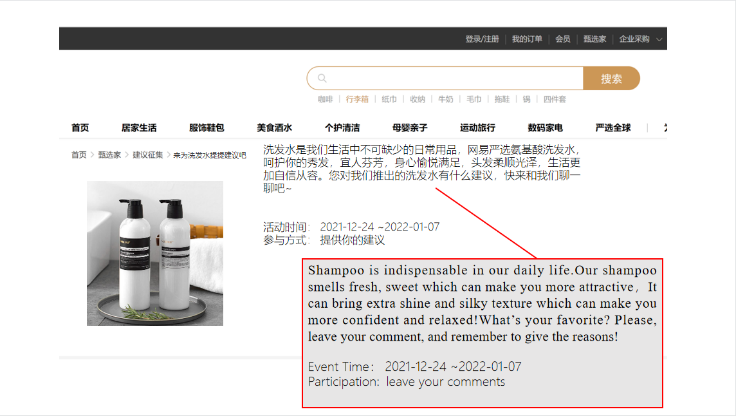


**Utilitarian—Affective Group**


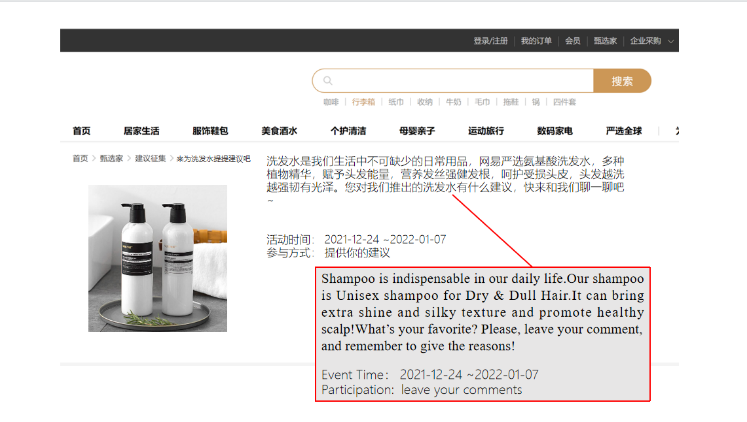


**Utilitarian—Cognitive Group**

**APPENDIX C. CONSTRUCT AND MEASUREMENT ITEMS**

| Construct | Measurement Items | Cronbach's α  (Study 2) | Cronbach's α  (Study 3) |
| --- | --- | --- | --- |
| Outcome simulation  Escalas and Luce (2004) | 1. Please indicate how much you thought about the end benefits or results of the shampoo while you were viewing the text. (not at all/very much)  2.While viewing the text, how much did you think about how you would feel after you had used the product? (not at all/very much) | 0.817 | 0.853 |
| Process simulation  Escalas and Luce (2004) | 1.While viewing the text, how much did you think about using the product on a daily basis? (not at all/very much)  2.“While viewing the text, how much did you think about the possibility of changing your current habits or behavior in order to use the product effectively? (not at all/very much)  3.While viewing the text, how much did you think about incorporating the product into your daily routine? (not at all/very much) | 0.822 | 0.839 |
| Participation intention  Lin et al. (2006) | 1. I plan to participate in this community activity in the future. (not at all/very much)  2. I intend to participate in this community activity in the future. (not at all/very much)  3. I expect to participate in this community activity in the future. (not at all/very much) | 0.866 | 0.814 |
